# RhCMV reactivation in SARS-CoV-2 infected aged rhesus macaques

**DOI:** 10.3389/fimmu.2025.1616490

**Published:** 2025-06-24

**Authors:** Jamin W. Roh, Peter A. Barry, Peter W. Hunt, Smita S. Iyer, Barbara L. Shacklett

**Affiliations:** ^1^ Graduate Group in Immunology, University of California, Davis, Davis, CA, United States; ^2^ Department of Medical Microbiology and Immunology, University of California, Davis, Davis, CA, United States; ^3^ California National Primate Research Center, Davis, Davis, CA, United States; ^4^ Division of Experimental Medicine, University of California, San Francisco, San Francisco, CA, United States; ^5^ Division of Infectious Diseases, Department of Medicine, UC Davis School of Medicine, Sacramento, CA, United States

**Keywords:** cytomegalovirus, rhesus macaque, SARS-CoV-2, animal models, viral reactivation

## Abstract

Human Cytomegalovirus (HCMV) is a ubiquitous virus with a global prevalence of 90%, but infection typically has minimal clinical impact in immunocompetent individuals. Consequently, most people are neither tested nor treated for HCMV. However, HCMV seropositivity is associated with higher hospitalization rates following SARS-CoV-2 infection compared to seronegative individuals, suggesting that viral reactivation may exacerbate severity of clinical symptoms. To investigate this, rhesus macaques naturally infected with rhesus cytomegalovirus (RhCMV) were experimentally inoculated with SARS-CoV-2 and monitored. Following inoculation, RhCMV viral loads in plasma increased from baseline, indicating reactivation. Within tissues, the lungs and ileum expressed immediate early protein-1 (IE1), a marker of active RhCMV infection. Additionally, elevated frequencies of circulating activated CD69^+^ memory T cells at day 3 suggested a recall response to a previously encountered pathogen. These findings demonstrate RhCMV reactivation and associated immune activation following SARS-CoV-2 infection, highlighting the rhesus macaque/RhCMV model as a valuable tool to elucidate the role of HCMV in SARS-CoV2 disease in immunocompetent hosts.

## Introduction

SARS-CoV-2 infection results in a spectrum of outcomes ranging from asymptomatic infection to death (1). Several factors have been linked to poor patient outcomes, including chronic conditions such as hypertension and type 2 diabetes, as well as viral coinfections (2–4). One such coinfection is human cytomegalovirus (HCMV), a beta herpesvirus with a global seroprevalence rate of >90% varying by region and increasing with age (5). HCMV is never cleared but instead persists in the immune host, periodically reactivating in response to inflammatory events such as acute viral infection (6). Although HCMV typically does not cause symptomatic disease in most healthy adults, seropositivity has been linked to increased risk of hospitalization following SARS-CoV-2 infection, indicating that HCMV reactivation may exacerbate the severity of SARS-CoV-2 disease (4). While mechanisms for HCMV’s contribution have not yet been identified, host responses to HCMV reactivation may be a driving factor for worsened patient outcomes. Intriguingly, HCMV elicits an unusually large population of virus-specific memory cells, with up to 40% of circulating memory T cells specific for HCMV (7), potentially resulting in greater inflammation due to a recall response against reactivated HCMV. In combination with the fact that HCMV can infect multiple cell types in every organ system (8), reactivation of HCMV and the corresponding recall response could potentiate inflammation and subsequent pathology (9). As more data are collected on SARS-CoV-2, there is increasing concern relating to neurologic, cardiac, and gastrointestinal outcomes as biopsies and autopsies of people with severe acute COVID-19 report SARS-CoV-2 RNA in multiple tissues (10, 11). These tissues are also sites of HCMV replication, as HCMV has been reported in cases of encephalitis, myocarditis, and gut dysbiosis (12–14). However, due to the critical state of many of these patients, biopsies are not readily available; therefore, an animal model to understand the impact of HCMV during SARS-CoV-2 infection is needed. The rhesus macaque serves as an excellent model for human HCMV infection using Rhesus CMV (RhCMV), a related herpesvirus with similar tropism and immunomodulatory properties. RhCMV reactivates in infected animals following coinfection with other pathogenic viruses, such as Simian Immunodeficiency Virus (SIVmac) (15). RhCMV is highly prevalent in both wild and breeding populations of macaques. Greater than 90% of wild rhesus macaques, and 100% of macaques in breeding cohorts have detectable antibodies specific to RhCMV (16) by 2 years of age (17, 18). Additionally, the rhesus macaque model recapitulates mild to moderate SARS-CoV-2 infection (19). This nonhuman primate model allows for investigation into tissues of concern to gain a better understanding of the complications that may arise from HCMV reactivation in tissues during SARS-CoV-2 infection.

## Materials and methods

### Rhesus macaques

Animal care and use protocols for this study were consistent with guidelines approved by the American Association for Accreditation of Laboratory Animal Care, the Animal Welfare Act, and the Institutional Animal Care and Use Committee at UC Davis. Eight Indian-origin rhesus macaques (*Macaca mulatta*) aged between 18 to 23 years were used for this work; all were housed at the California National Primate Research Center (CNPRC). All animals prior to the study tested positive for RhCMV via antibody titer testing; additionally, all animals were diagnosed as diabetic and received continuing treatment throughout the duration of the study as previously reported (19).

### Clinical assessments

Clinical assessments were performed by a board-certified laboratory animal veterinarian throughout the duration of the study. Responsiveness, mucosal discharge, respiration and stool consistency were assessed and reported as cage side clinical scores ranging from 0 to 15, with higher numbers indicating greater symptom severity, as described in the rubric shown in **Supplementary Figure S1A**.

### Animal treatments and infections

**Figure 1:**
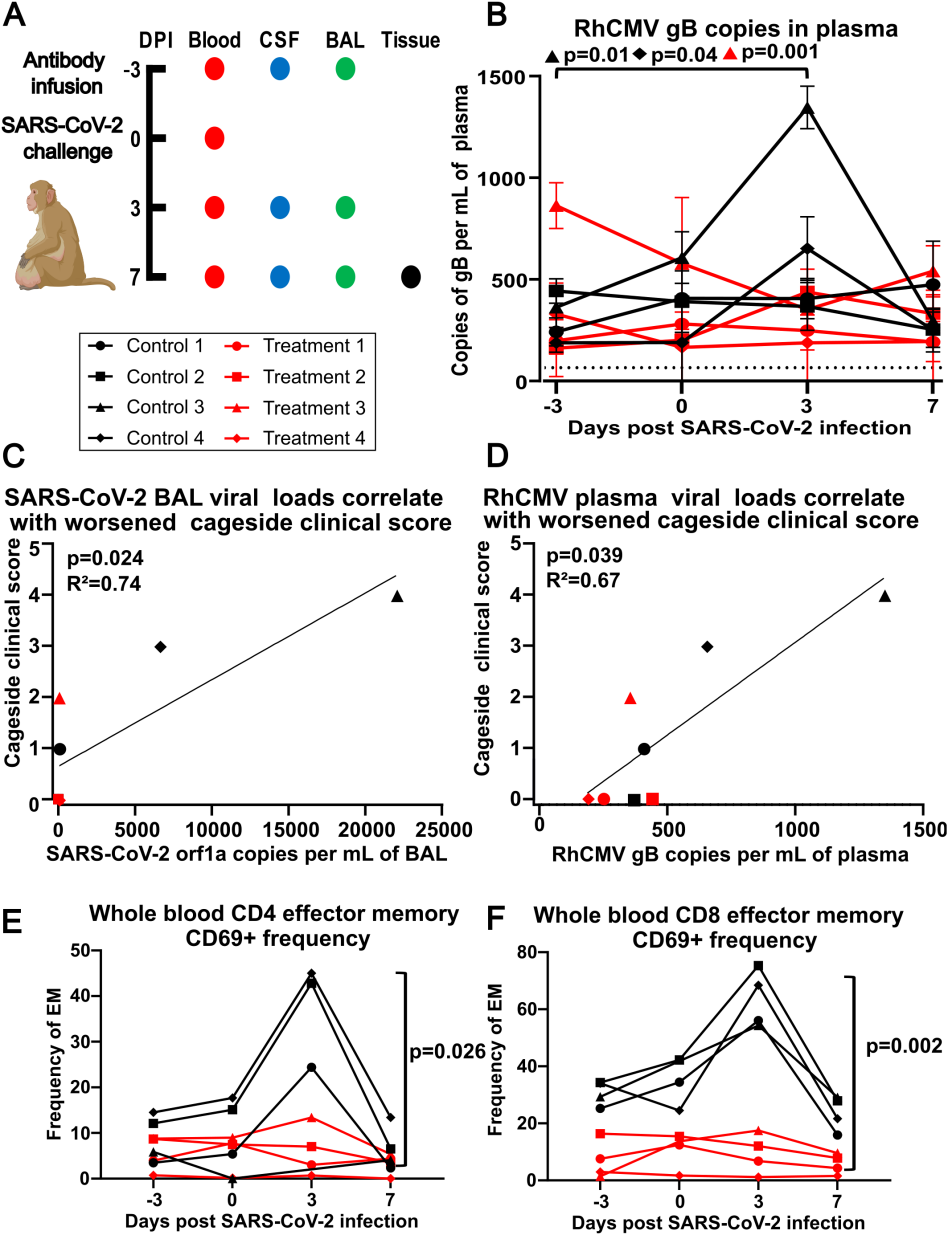
**(A)** Experimental design. Dots indicate days a particular sample was collected (DPI – days post infection, CSF - cerebrospinal fluid, BAL - bronchoalveolar lavage). Animals were given either control antibody 3BNC117 (targeting HIV GP120) or treatment antibodies C114-LS (IC_50_ 2.55 ng/ml) and C135-LS (IC_50_ 2.98 ng/ml) (1:1 ratio), which are neutralizing antibodies against SARS-CoV-2 strain Wuhan-Hu-1, dosed at 20 mg/kg intravenously at 2 mL per minute at -3 DPI. All 8 animals were infected intratracheally (2.5mL 2.5x10^6^ PFU) and intranasally (0.5mL 2.5x10^6^ PFU) with SARS-CoV-2. Animals were euthanized at +7 DPI. **(B)** RhCMV plasma viral loads. The y-axis shows RhCMV gB DNA copies per mL of plasma at times indicated on the x-axis. Dotted line indicates the limit of detection at 40 copies per mL of plasma. Using Dunnett’s multiple comparison test, Control Animal 3 (p=0.01) and Control Animal 4 (p=0.04) both had statistically significant increases to RhCMV gB in plasma, while Treatment Animal 3 (p=0.001) had a statistically significant decrease to RhCMV gB in plasma at 3 DPI compared to -3 DPI. **(C)** Correlation plot of SARS-CoV-2 orf1a RNA from BAL at 3 DPI relative to cage side clinical scoring at 3 DPI using a one-tailed Spearman’s rank correlation coefficient, p=0.024 and R^2^ = 0.74. **(D)** Correlation plot of RhCMV gB DNA copies per mL of plasma at 3 DPI relative to cage side clinical scoring at 3 DPI using a one-tailed Spearman’s rank correlation coefficient, p=0.039 and R^2^ = 0.67. **(E)** The control group had significantly increased activation compared to the treatment group at day 3 (p=0.026) in CD4 T cells using a mixed model analysis with a Greenhouse-Geisser correction and Tukey’s multiple comparison test. **(F)** CD8 T cells had significant increases across all time points: day -3 (p=0.002), day 0 (p=0.025), day 3 (p=0.0002), and day 7 (p=0.004) using a mixed model analysis with a Greenhouse-Geisser correction and Tukey’s multiple comparison test (significance bars not shown for days -3, 0, and 7 for figure clarity).

Details of antibody treatments and viral inoculations were previously described in a study designed to test the ability of neutralizing monoclonal antibodies directed against SARS-CoV-2 to limit adverse inflammatory consequences of SARS-CoV-2 exposure in rhesus macaques (19). An overview of the study design is shown in **Figure 1A**. Briefly, 4 control macaques were given antibody 3BNC117 targeting the binding site of HIV gp120 (used as a negative control, as it does not target SARS-CoV-2), while the 4 treated animals were given both C114-LS (IC_50_ 2.55 ng/ml) and C135-LS (IC_50_ 2.98 ng/ml) (1:1 ratio) neutralizing antibodies directed against SARS-CoV-2 strain Wuhan-Hu-1 (GenBank: NC_045512). These antibodies were administered on day -3 of the study at a concentration of 20 mg/kg delivered intravenously at 2mL/minute. At day 0, all 8 animals were infected intratracheally (2.5mL 2.5x10^6^ PFU) and intranasally (0.5mL 2.5x10^6^ PFU) with SARS-CoV-2 from BEI Resources (SARS-CoV-2–2019 nCoV/USA WA1/2020; NR 52352; Lot/Batch #70033952).

### Specimen collection and processing

Details of collection and processing were previously described (19). Briefly, animals were anesthetized with 10 mg/kg of ketamine. EDTA tubes were used to collect blood at days -3, 0, +3, and 7. Cerebrospinal fluid (CSF) was aspirated up to 2mL or 0.5mL/kg on days 0, 3, and 7. RhCMV viral loads were determined using CSF supernatant. Tissues collected at necropsy (brain, heart, and lung) were mechanically processed using scissors, and DNA was isolated using standard phenol/chloroform extraction.

### Quantitative polymerase chain reaction

DNA isolated from phenol chloroform extract was quantified, then used against a known standard. Taqman master mix was used with primer probe sequences “ACATCTGGCCGTTCAAAAAAAC” and “TGCGTACTATGGAAGAGACAATGC”, and 5’-TET-CCAGAAGTTGCGCATCCGCTTGT-TAMRA-3’ targeting gB DNA in RhCMV. Samples were run on either a QuantStudioFlex 6 or 7 (ThermoFisher Scientific).

### Immunohistochemistry

Tissues were fixed in 4% paraformaldehyde and then embedded in paraffin. Slides were cut at 4 microns and rehydrated using xylene, ethanol and water. Antigen retrieval (Biocare Medical SKU: CB910M) was done using a pressure cooker (Instant Pot UPC: 859716007205; blocking reagent was Agilent Cat. No. X0909). Staining was done with a non-commercially available rabbit polyclonal antiserum recognizing the RhCMV IE1 protein (exon 4). Antibody was visualized using 3,3’ Diaminobenzidine (DAB) Substrate Kit (Vector SK-4011) and Elite ABC Kit, Peroxidase (VECTASTAIN PK-6100).

### Flow cytometry

Cells were analyzed fresh as previously described (19) using BD Symphony and BD Fortessa flow cytometers located at the CNPRC. The following antibodies were used: CD3 AF700 (Cat. No. 557917), CD4 BV540 (Cat. No. 563737), CD8 BUV805 (Cat. No. 564913), and CD95 BUV737 (Cat. No. 564710), all from BD Biosciences; and CD20 APC/Cy7 (Cat. No. 302314), CD28 PE/Dazzle™ 594 (Cat. No. 302942), and CD69 BV711 (Cat. No. 310944), all from BioLegend.

### Statistics

Correlations between cage side clinical scoring and viral load data (SARS-CoV2 and RhCMV) were analyzed using Spearman’s rank correlation coefficient. Changes in viral load over time were assessed using Dunnett’s multiple comparison test with a Greenhouse-Geisser correction, with -3 DPI (days post-infection) as the control time point. For each tissue, RhCMV gB viral loads were compared by treatment type using Mann-Whitney tests; no statistically significant differences were found. Flow cytometry data were analyzed using mixed model analysis with a Greenhouse-Geisser correction and Tukey multiple comparison test.

## Results

### RhCMV viral loads in plasma correlate with worse clinical outcomes

Eight female RhCMV-positive rhesus macaques, aged 18–23 years, were experimentally dosed with SARS-CoV-2 intratracheally and intranasally and monitored for 7 days post infection. Four of the animals were pre-treated with SARS-CoV-2 monoclonal antibodies (mAB) C114-LS and C135-LS as previously described (19), and the remaining 4 were treated with a control antibody 3 days prior to inoculation. Blood was collected on days -3, 0, + 3, and +7. Cerebrospinal fluid (CSF) and bronchoalveolar lavage (BAL) were collected on days -3, +3, and +7. Brain, colon, ileum, heart, and lung tissues were collected on day +7 during necropsy (**Figure 1A**).

RhCMV plasma viral loads, measured using RhCMV gB to quantify viral DNA load, reached up to 1,400 copies per mL (**Figure 1B**). Following SARS-CoV-2 infection, two (25%) of the animals had statistically significant increases in plasma RhCMV from baseline measurements as detected by qPCR, while one had a statistically significant decrease (**Figure 1B**). Interestingly, the two animals with increased plasma RhCMV were in the group that received control mAb (Control 3 and Control 4). While cage side clinical scoring at day 3 was positively correlated with SARS-CoV-2 viral load in bronchoalveolar lavage (**Figure 1C**; **Supplementary Figures S1A, B**), analysis of RhCMV plasma viral load and cage side clinical scoring at day 3 indicated greater clinical disease was associated with RhCMV DNA in plasma (**Figure 1D**). SARS-CoV-2 viral loads and kinetics were originally reported by Verma and colleagues [see reference (19), **Figure 1D**]. Notably, the animals that had significant increases in RhCMV plasma viral load had the highest clinical scores on day 3 and had not received SARS-CoV-2 mAb.

### Activation of circulating effector memory T cells following SARS-CoV-2

We investigated T-cell activation following SARS-CoV-2 infection and found that a circulating effector memory T-cell population, defined as CD3^+^CD28^-^CD95^+^ in both CD4^+^ and CD8^+^ subsets and expressing CD69, a marker of recent activation, was transiently increased at day 3 and contracted by day 7 prior to SARS-CoV-2 viral clearance (**Figures 1E, F**; **Supplementary Figures S1C**) (19).

### Pulmonary observations: RhCMV found in lungs post SARS-CoV-2 infection

**Figure 2:**
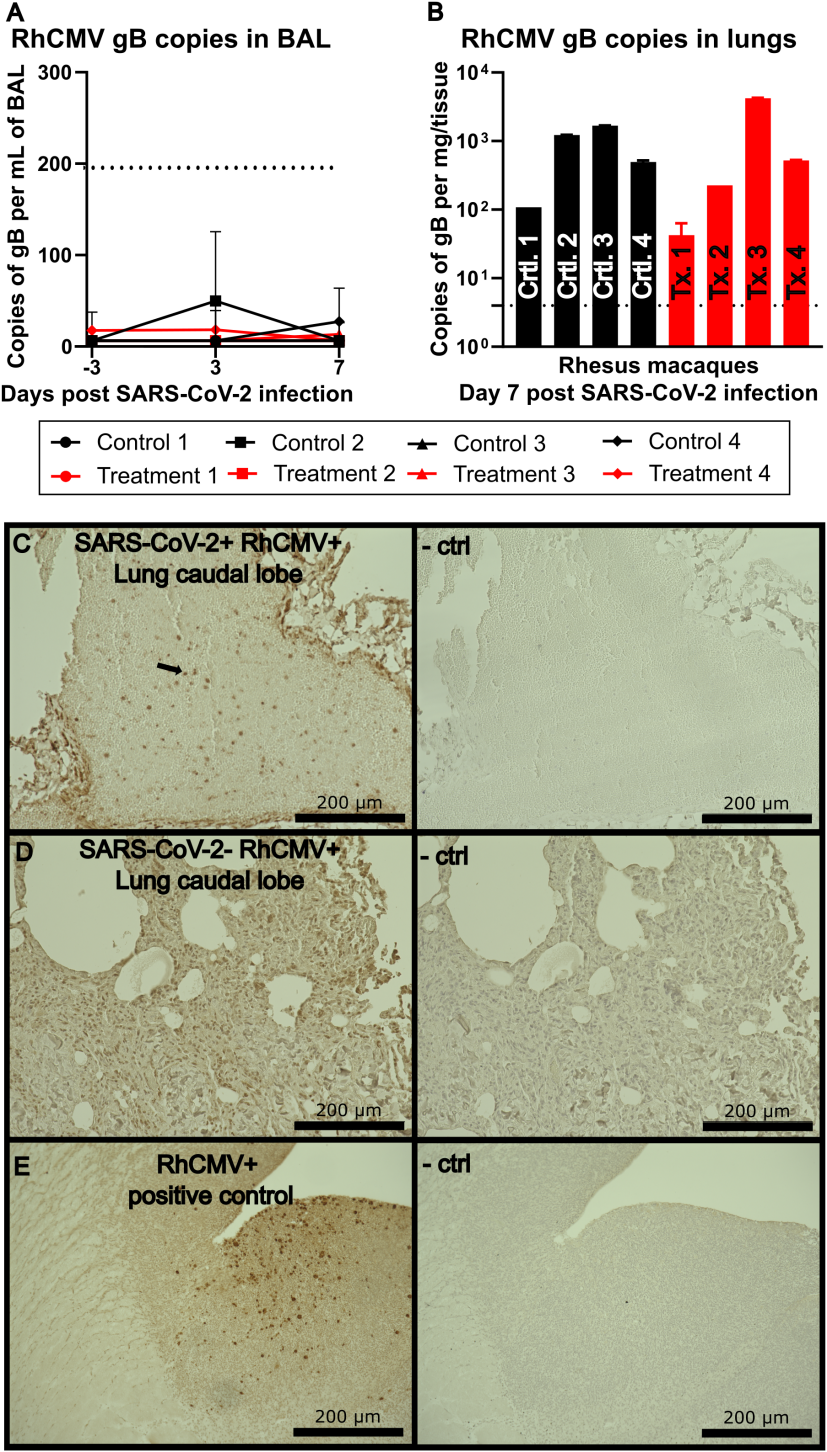
**(A)** RhCMV viral loads isolated from the BAL targeted against gB. The dotted line indicates limit of detection at 200 copies per mL of isolated fluid. **(B)** RhCMV viral load isolated from lung tissue targeted against gB. **(C)** IHC of lung caudal lobe stained with a non-commercially available rabbit polyclonal antiserum against exon 4 of RhCMV IE1 and visualized using 3,3’-diaminobenzidine (DAB) (brown) chromogen, representative image. Right image shows a serial section stained without IE1 polyclonal antiserum and used as a negative control. **(D)** Representative IHC of lung caudal lobe of an animal not infected with SARS-CoV-2 but positive for RhCMV stained with a non-commercially available rabbit polyclonal antiserum against exon 4 of RhCMV IE1, and visualized using DAB (brown) chromogen, representative image. Right image shows a serial section stained without IE1 polyclonal antiserum and used as a negative control. **(E)** RhCMV^+^ known positive control as a reference for IHC staining, visualized using DAB (brown) chromogen. Right image shows a serial section stained with IE1 polyclonal antiserum as a negative control.

Our initial investigation included testing for the presence of RhCMV in the pulmonary compartment. Analysis of BAL fluid indicated no detectable RhCMV by qPCR (**Figure 2A**). To detect reactivation of RhCMV in the lung tissue, caudal lobe samples collected at 7 days post infection were analyzed by qPCR for gB viral DNA expression and by IHC for IE1 protein expression. These proteins have been previously described as necessary for viral entry and as a marker for RhCMV reactivation, respectively (20, 21). RhCMV gB was detected in all 8 animals with viral loads up to 4,000 copies per mg of the caudal lobe (**Figure 2B**). IE1 staining of the lungs revealed positive staining in the vasculature rather than within the lung parenchyma in most cases, suggesting that reactivated RhCMV may be originating from the circulation rather than the lung itself (**Figure 2C**). RhCMV positive, SARS-CoV-2 negative animals were used as a negative control in addition to tissue stained without primary antibody (**Figure 2D**). A previously confirmed RhCMV^+^ sample was used as a positive staining control (**Figure 2E**).

### Limited cardiac involvement by RhCMV in SARS-CoV-2 infection

**Figure 3:**
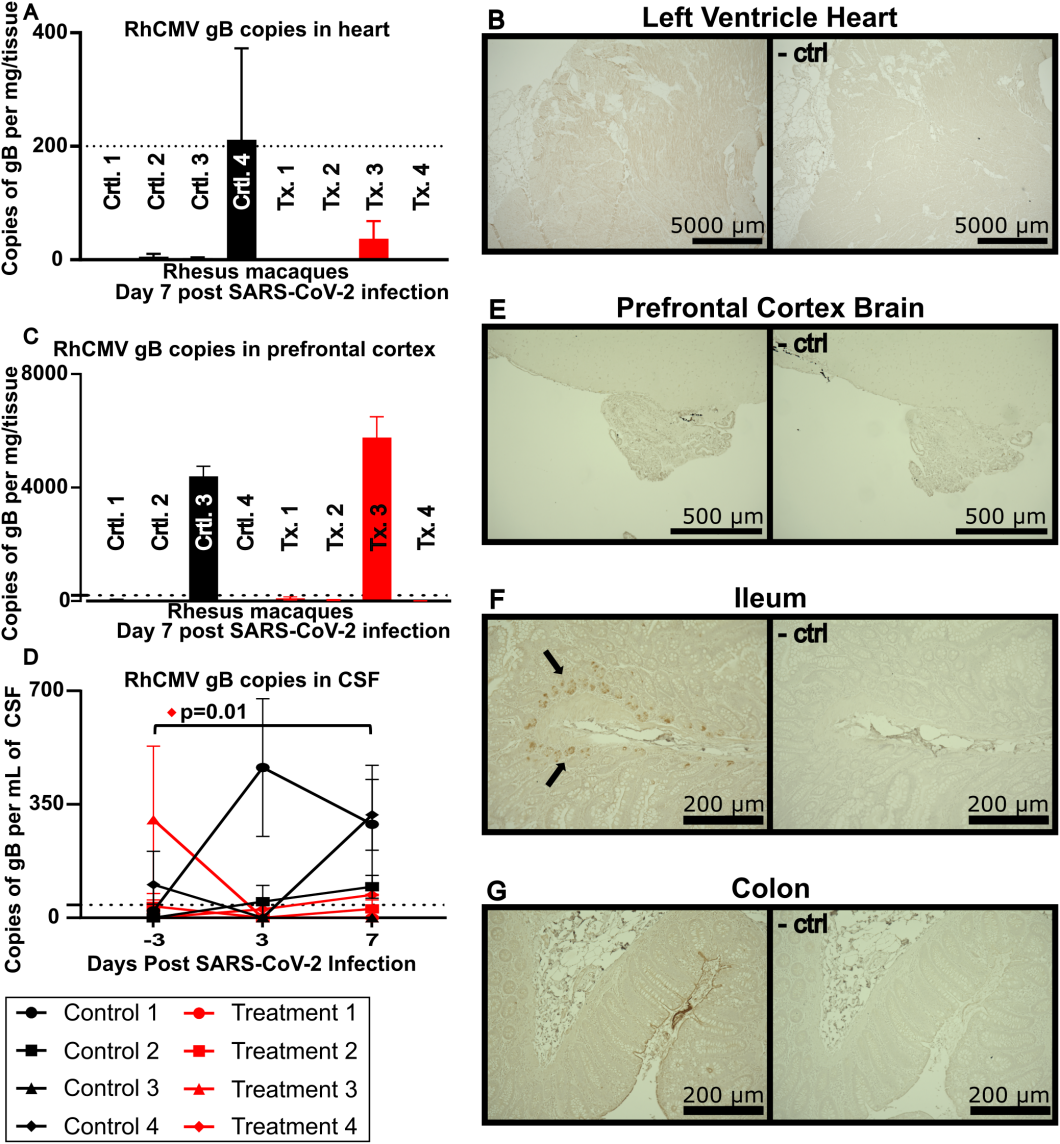
**(A)** RhCMV viral loads in heart tissue. The dotted line indicates limit of detection at 200 copies per mg of heart tissue. **(B)** IHC of left ventricle of the heart stained with a non-commercially available rabbit polyclonal antiserum against exon 4 of RhCMV IE1 and visualized using DAB (brown) chromogen, representative image. Lower image shows a serial section stained without IE1 polyclonal antiserum and used as a negative control. **(C)** RhCMV viral loads in the prefrontal cortex of the brain targeted against gB. The dotted line indicates limit of detection at 200 copies per mg of tissue. **(D)** RhCMV viral load in CSF reported as gB copies/mL. Dotted line indicates limit of detection at 40 copies per mL of CSF. Using Dunnett’s multiple comparison, Treatment Animal 4 had a statistically significant increase in RhCMV gB in the CSF. **(E)** IHC of prefrontal cortex of the brain **(E)**, ileum **(F)**, and colon **(G)**, stained with a non-commercially available rabbit polyclonal antiserum against exon 4 of RhCMV IE1, representative image. Right image shows a serial section stained without IE1 polyclonal antiserum and used as a negative control.

Given the known complications of SARS-CoV-2 infection in nonprimary tissues such as brain/CNS, gastrointestinal tract and heart/myocardium, we next investigated tissues that can serve as reservoirs of HCMV with the potential for infection with SARS-CoV-2. In the animals in this study, we were unable to detect any levels of RhCMV in heart tissue by qPCR or IHC. Accordingly, it appears likely that this cohort did not include animals with RhCMV reactivated in the heart (**Figures 3A, B**).

### Central nervous system involvement

In contrast to findings in the heart, our investigations of the CNS indicated reactivation of RhCMV. Two animals showed RhCMV gB expression in the prefrontal cortex (**Figure 3C**), and 3 animals had detectable levels of RhCMV gB expression by day 7 in the CSF (**Figure 3D**). Three of the 4 animals that had detectable RhCMV in the CSF and/or brain had not received the SARS-CoV-2 mAb. No RhCMV was detected by IHC when staining tissue from the prefrontal cortex for IE1 protein (**Figure 3E**).

### Gastrointestinal tissue involvement

As ACE2, the receptor for SARS-CoV-2, is also present in the GI tract, gut tissue samples were analyzed to investigate the reactivation of RhCMV (22). Ileum and colon samples were stained for CMV IE1 protein by IHC and revealed IE1 expression localized to ileal crypts in 4 of the 8 animals studied (**Figure 3F** and data not shown). In contrast, no detectable RhCMV IE1 protein was found by IHC in colon tissue (**Figure 3G**). Only the 4 animals that received control antibody, but not those that received SARS-CoV-2 monoclonal antibody, showed RhCMV expression in terminal ileum.

## Discussion

As SARS-CoV-2 continues to infect humans worldwide, it remains important to understand the factors that contribute to poor clinical outcomes, especially if they can be prevented. While HCMV rarely causes symptomatic disease outside of congenitally infected infants and immunocompromised individuals, HCMV seropositivity is correlated with poor patient outcomes, most notably of those in critical care (4, 23). In this single-experiment exploratory study, which comes with inherent limitations in robustness and reproducibility, we used the rhesus macaque/RhCMV model to investigate the impact of persistent CMV infection on SARS-CoV-2 disease.

Our results revealed an association between RhCMV detection in plasma and worse clinical outcomes. Two of the eight animals in this study (25%), exhibited significant increases in detectable RhCMV mRNA expression after SARS-CoV2 infection; both were in the group that was not treated with SARS-CoV2 mAb. These findings align with published observations made in the clinic, where 20.5% of SARS-CoV-2 patients had evidence of CMV reactivation in plasma (24). Furthermore, in our study, RhCMV plasma viral load was positively correlated with cage side clinical scoring at day 3, suggesting a relationship between HCMV activation and more severe clinical disease, and coincided with effector memory T-cell activation and subsequent clearance. This finding appears to recapitulate a recent study of HCMV reactivation in SARS CoV-2 patients with severe CoV-2 disease (25). However, in the absence of antigen-specific assays, we cannot rule out either a memory T-cell response to previous coronavirus exposures, and/or cytokine-driven “bystander” T-cell activation.

The lungs serve as the primary site of infection and pathogenesis for SARS-CoV-2 as well as a potential reservoir for HCMV. Analysis of BAL fluid in our study animals showed no detectable RhCMV by qPCR. This was consistent with previously published clinical data which revealed that BAL was an infrequent site of HCMV detection in human SARS-CoV2 infection (26). However, in our study, caudal lobe samples collected at 7 days post infection from all 8 animals, including those treated with SARS-CoV-2 antibody, were positive for RhCMV gB expression by qPCR, with viral loads as high as 4,000 copies per mg of tissue. We also detected RhCMV IE1 protein expression in lung tissue by immunohistochemistry, particularly associated with the vasculature. Taken together, these findings demonstrate RhCMV reactivation in lung tissue of macaques following SARS-CoV-2 infection.

SARS-CoV-2 has been found in cardiac tissues in 60% of autopsy patients (27). In addition, HCMV is known to infect the heart and can induce scarring of cardiac tissue (12). However, in the animals in this study we were unable to detect any levels of RhCMV in heart tissue by qPCR or IHC. Human studies have shown associations between HCMV and myocarditis; however, these are considered rare events in immune competent individuals (28, 29).

In contrast to our findings in heart tissue, investigations of the CNS indicated expression of RhCMV in 4 animals in either the prefrontal cortex and/or CSF. Three of the 4 positive animals had not been treated with SARS-CoV-2 antibodies. The amounts of RhCMV detected in the CSF of these animals were lower than viral loads typically observed in humans with HCMV encephalitis during SARS-CoV-2 infection, as outlined in a previous case study (30), despite one animal (#4 in the treatment group) showing a statistically significant increase in RhCMV gB copies (p=0.01). The differences in expression of viral gB DNA copies measured by qPCR and viral IE1 protein measured by IHC could be due to viral latency, localization within the brain, or possibly to trafficking of rare RhCMV-infected cells into the brain without active infection. Interestingly, the animal with the highest plasma viral load (control animal #3) also had high copy numbers of RhCMV gB in the prefrontal cortex. While this may indicate a reactivated reservoir, it is also possible that increased blood brain barrier permeability caused by SARS-CoV-2 allowed for reactivated circulating RhCMV found in plasma to better traffic into the brain. Several reports have implicated a link between SARS-CoV-2 infection and blood-brain barrier disruption (31–33).

HCMV detection in the CSF does not appear to be a common finding during SARS-CoV-2 in clinical settings (34, 35). While there have been reports documenting the presence of RhCMV in the brain, to our knowledge there have been no detailed studies of RhCMV viral load in the CSF of adult rhesus macaques, although HCMV is often measured in CSF to determine CNS involvement in humans (36). HCMV has been known to cause cognitive impairment in infants infected *in utero* and can induce encephalitis in patients with suppressed immune systems such as those living with HIV-1 or who have undergone transplants and are iatrogenically immunosuppressed (37). With reports of neurological symptoms in people with typical SARS-CoV-2 infection as well as Long COVID, the rhesus macaque model may be useful in understanding the neurological complications of these diseases. Intriguingly, there are also reports suggesting that HCMV seropositivity may be protective against neurological damage from Long COVID (38).

Gastrointestinal distress frequently arises in SARS-CoV-2 infection (39, 40). HCMV infection has been linked to intestinal epithelial barrier dysfunction in the setting of HIV, potentially contributing to microbial translocation and chronic inflammation (41). Because the SARS-CoV-2 receptor, ACE2, is expressed in the GI tract, we examined gut tissue samples for expression of RhCMV (22). The observation that the four animals treated with control antibody showed RhCMV expression in terminal ileum, while the animals treated with SARS-CoV-2 monoclonal antibody did not, suggests reactivation of RhCMV secondary to SARS-CoV-2 expression in this tissue.

The presence of RhCMV antigen in the lungs following SARS-CoV2 infection suggests RhCMV reactivation, as RhCMV antigen is typically absent from the lungs of otherwise healthy animals with latent RhCMV. RhCMV reactivation could occur as a consequence of IL-6 induction during SARS-CoV-2 infection (42), as observed in other rhesus macaque studies (43) and in this specific cohort as well (19). IL-6 has previously been shown to induce expression of IE1 (44), which is a marker of HCMV reactivation. Additionally, mouse models have shown that cytokines can induce pathology following murine CMV reactivation, though the mechanisms by which the virus causes pathology have not yet been fully elucidated (45).

HCMV expression can induce proinflammatory cytokines as well as prevent effective immune cell trafficking through immunomodulatory proteins (46–48). These factors in combination could lead to worsened pathology. On the other hand, as noted above, a study of Long COVID has found that HCMV seropositivity is linked to decreased neurological impairment, potentially related to expression of the HCMV-encoded vIL-10 which may suppress inflammation (38). While CMV was discussed in this paper, other *Herpesviridae* such as Epstein Barr Virus/Human Herpesvirus 4 (HHV4), Human Herpesvirus 6 (HHV6), and Human Herpesvirus 8 (HHV8) may also reactivate, contributing to worse clinical outcomes in patients with COVID-19 (49). Additional studies will be required to further clarify these findings. The rhesus macaque model can also be utilized to investigate the early use of anti-herpesvirus drugs such as valganciclovir to determine the specific effects of RhCMV reactivation on SARS-CoV2 disease. Because HCMV has been linked to worsened outcomes not only in SARS-CoV-2 but other infectious diseases as well, early HCMV testing and antivirals may lead to improved patient outcomes.

## Data Availability

The original contributions presented in the study are included in the article/**Supplementary Material**. Further inquiries can be directed to the corresponding authors.

## Supplementary material

Supplementary Figure 1
**(A)** criteria used for cage side scoring of rhesus macaques, where severity of disease was ranked as follows: no clinical disease (0-4), mild (5-9), moderate (10-15), severe (>15). **(B)** summary of cage side clinical scoring at 3 days post SARS-CoV-2 infection. **(C)** Flow cytometry gating strategy used to identify CD69^+^ effector memory CD4 and CD8 T cells.
