# Supplementary figures and images for: RhCMV reactivation in SARS-CoV-2 infected aged rhesus macaques
Source: Front Immunol. 2025 Jun 24;16:1616490. doi: 10.3389/fimmu.2025.1616490 (PMC12234489; doi:10.3389/fimmu.2025.1616490)

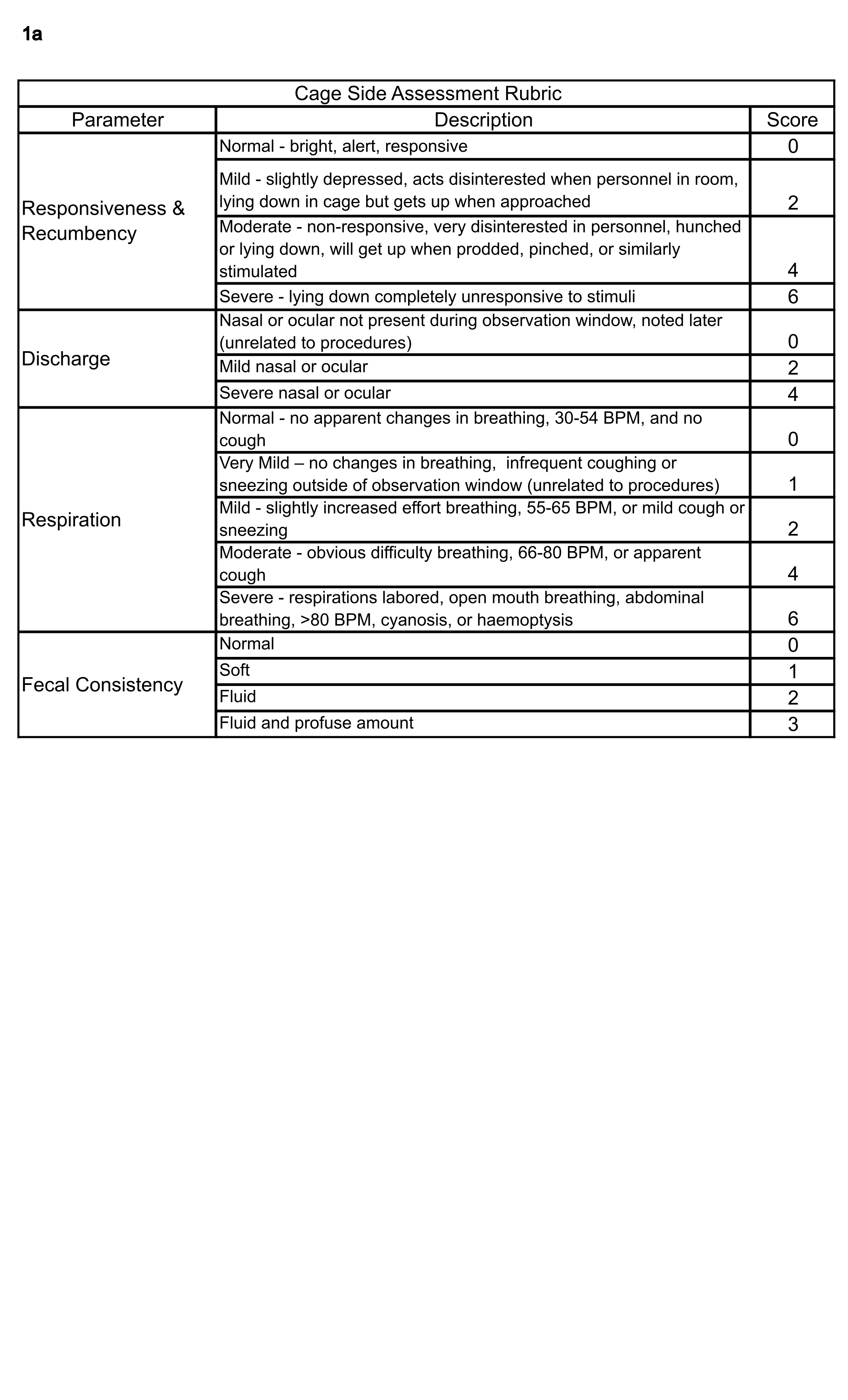

Supplement: Supplementary Figure 1 — (A) criteria used for cage side scoring of rhesus macaques, where severity of disease was ranked as follows: no clinical disease (0-4), mild (5-9), moderate (10-15), severe (>15). (B) summary of cage side clinical scoring at 3 days post SARS-CoV-2 infection. (C) Flow cytometry gating strategy used to identify CD69+ effector memory CD4 and CD8 T cells. [file Image1.tiff]

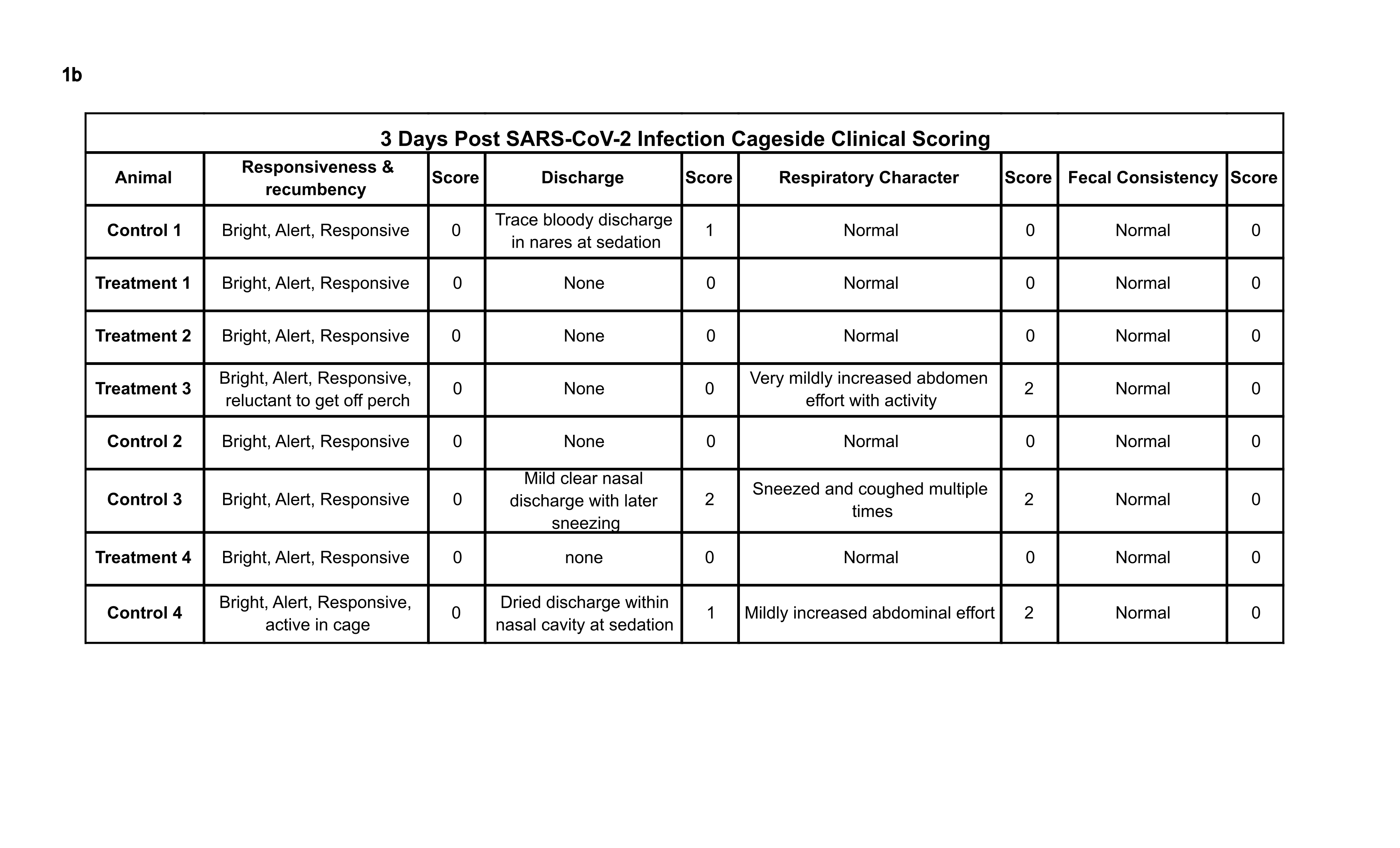

Supplement: Supplementary file 2 [file Image2.tiff]

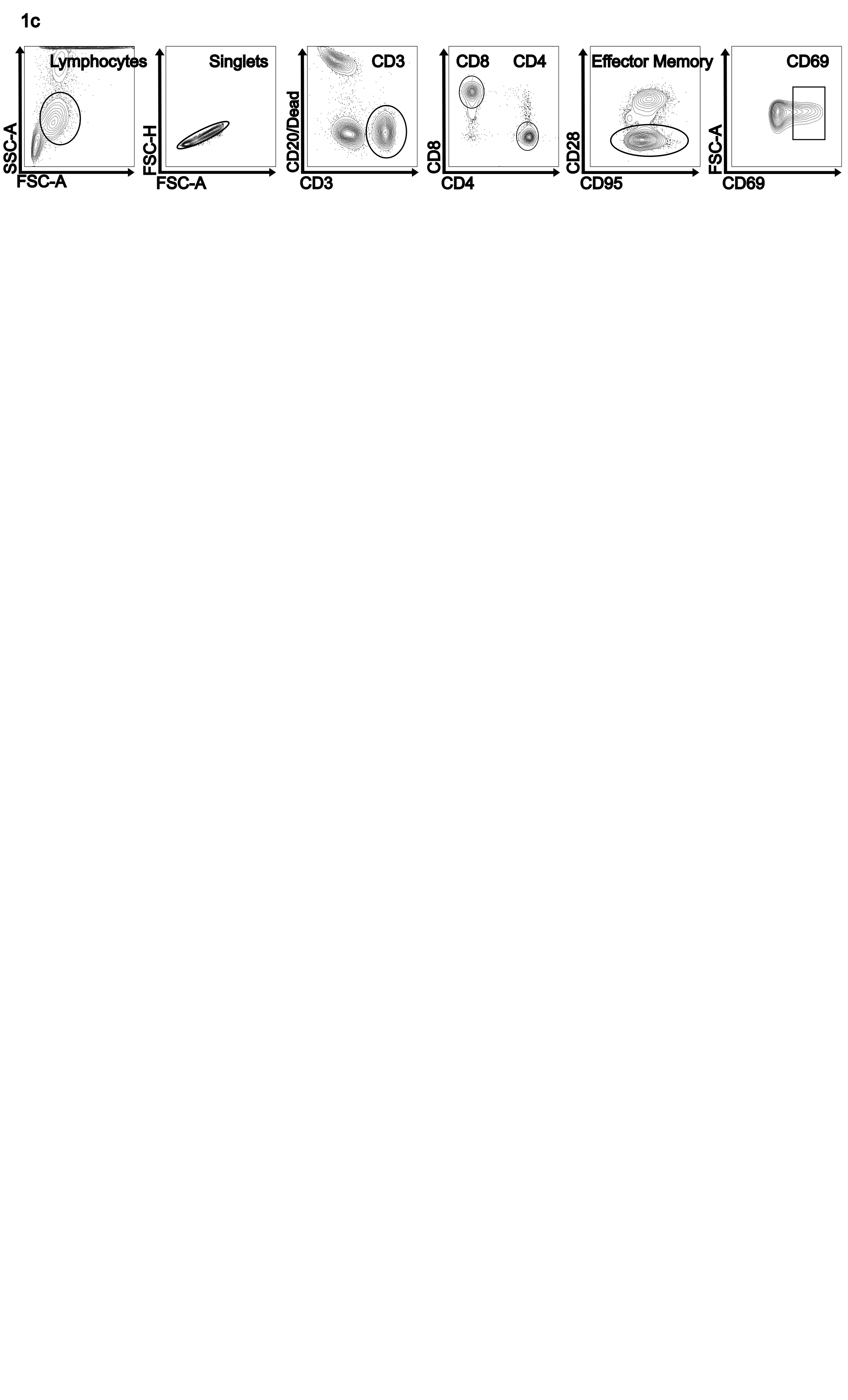

Supplement: Supplementary file 3 [file Image3.tiff]
